# Generation of alveolar organoids from human induced pluripotent stem cells using a stirred-tank bioreactor

**DOI:** 10.3389/fbioe.2026.1858660

**Published:** 2026-08-05

**Authors:** Bettina Budeus, Lea Cremer, Zehra Fatma Sevindik, Diana Klein

**Affiliations:** Institute for Cell Biology (Cancer Research), Medical Faculty, University of Duisburg-Essen, Essen, Germany

**Keywords:** 3D culture, bioengineering, bioreactor, IPSC, lung organoids

## Abstract

**Introduction:**

Organoids are increasingly establishing themselves as a valuable, patient- centered alternative to preclinical animal models as they are highly complex models for organ development studies and, in particular, robust disease models for mechanistic investigations and therapy response assessments.

**Methods:**

We previously established a very simple and practical protocol for producing induced pluripotent stem cell-derived lung organoids (iPSC-LuOrgs) from pre-differentiated embryoid bodies using a stirred bioreactor system. Here we adapted the protocol to investigate the generation of lung organoids directly from iPSCs in stirred suspension, and performed a comprehensive cellular and molecular characterization, including single-cell RNA sequencing, of the resulting LuOrgs.

**Results:**

Direct transfer of iPSCs as a single-cell suspension into the bioreactor and subsequent differentiation led to the formation of lung structures that appeared less differentiated and were predominantly alveolar in nature. Fibroblasts and bronchial epithelial cells were generated only to a limited extent.

**Discussion:**

Pre- differentiation of iPSCs, e.g., in embryoid bodies seems to be a necessary prerequisite for the efficient generation of iPSC-LuOrgs, which then exhibit alveolar and bronchial cell types and structures supported by mesenchymal cells.

## Introduction

Accurate replication of mature human tissues needs complex tissue culture systems. In recent years, organoids have emerged as a valuable, patient-centered alternative to preclinical animal models in human developmental research, disease modeling, and cancer research (Drost and Clevers, 2018; Verstegen et al., 2025). The organoids generated here from induced pluripotent stem cells (iPSCs) have proven particularly valuable among the advanced organoid platforms capable of more precisely replicating the cellular architecture of the lung’s airways and alveoli (Xu et al., 2023; Tamai et al., 2022; Vazquez-Armendariz and Tata, 2023). These lung organoids, in addition to the (patient-centered) modeling of lung diseases (Strikoudis et al., 2019; Leibel et al., 2020; Miller et al., 2019), also facilitate the testing of potential therapeutic substances for the development of new drugs (Dutta et al., 2017; Li et al., 2019a). Many potential organoid applications however, such as high-throughput drug screening, require very high cell or organoid numbers. Cell-based therapies, in turn, require highly mature organoids. Bioreactor technology offers new possibilities for conveniently and controllably scaling up an established manufacturing process (Tam et al., 2022; Unagolla and Jayasuriya, 2022).

Commercial bioreactors, designed for the production of biological materials from living cells and organisms, typically consist of large culture vessels (tanks) equipped with a stirring mechanism and, if necessary, a pump for moving liquids in and out of the culture vessels. Environmental conditions such as pH, dissolved gas concentration, flow/stirring rates, and nutrient content within the system can be precisely controlled (Wang et al., 2005; Nikita et al., 2023). Furthermore, bioreactors are increasingly being used to automate and precisely control the three-dimensional culture of cells and tissues *in vitro* in order to improve the production of these cultures (Borys et al., 2021; Licata et al., 2023). Based on the high costs of cell culture media and additives, bioreactors for organoid culture are usually smaller, meaning they require much less medium so that these (initially) reduced dimensions can be used for targeted optimizations regarding oxygen and nutrient concentrations, shear forces, or flow rates (Licata et al., 2023; Phelan et al., 2019).

We recently developed a relatively simple and practical method for generating free-floating lung organoids from human iPSCs (iPSC-LuOrgs) using ultra-low attachment plates, thus eliminating the need for a matrix (Budeus et al., 2024; Budeus et al., 2025a). Herein, iPSCs were initially cultured as embryoid bodies (EBs) before being further cultured with differentiation medium, ultimately leading to the differentiation of typical lung epithelial and mesenchymal cells, arranged in a well-defined lung structure. Combination with radiotherapy was then used to demonstrate that this LuOrg model reproduces *in vitro* many features of radiation-induced lung injury (RILI) observed in patients (Budeus et al., 2024). Subsequently this protocol was used to perform the large-scale differentiation of pre-differentiated EBs into lung organoids under controlled conditions in a bioreactor (Budeus et al., 2025b; Van Heuve et al., 2025). This yielded organoids of the same high quality as the LuOrgs obtained using the manual method, i.e., cultivation in ultra-low attachment plates (Budeus et al., 2025b). Here, we investigated whether lung organoids can also be formed by directly transferring expanded iPSCs into the bioreactor and then initiating the differentiation process. This would represent a further step towards fully automated lung organoid generation from iPSCs.

## Materials and methods

### iPSC expansion and bioreactor cultivation

Two commercially available human iPSC lines were used. The SCTi003 iPSC cell line (iPSC #1) purchased from StemCell Technologies (Vancouver, BC, Canada), and the iPS01 iPSC cell line (iPSC #2) from ALSTEM Inc. (Richmond, CA, United States). Human iPSCs were classically maintained and expanded on Vitronectin XF™-coated culture dishes in mTeSR1 medium (#15883465, StemCell Technologies, Vancouver, BC, Canada) according to the manufacture’s protocols under standard cell culture conditions at 37 °C, 5% CO_2_ in humidified atmosphere. Cells were monthly tested for *mycoplasma* contamination. Expanded iPSCs were harvested using an enzyme-free cell dissociation reagent (#100–0485, StemCell Technologies), counted, and subsequently transferred (in normal growth medium, NGM, mTeSR1 medium) with 6.250 cells/mL (total volume 200 mL) to the pre-equilibrated culture vessel of the bioreactor (Comfy Cell bioreactor, BioThrust, Aachen, Germany) using the following parameters: DO set point 95%; oxygen transfer rate 0.6 (0.8) mmol/L✕h; pH range 7.2–7.5; sparger gassing 0.5 L/min constant flow, air +5% CO_2_ on demand, open-loop off-gas circulation and 80 rpm stirring). After 4–5 days, half of the medium was replaced by 100 mL Branching Lung Organoid Medium [BLO-Medium: DMEM F12 with GlutaMAX supplemented with1× N-2, 1× B27, 1× penicillin–streptomycin (5,000 U/mL), 0.4% (vol/vol) BSA, 0.4 µM monothioglycerol, 50 μg/mL ascorbic acid, 10 ng/mL KGF/FGF7, 10 ng/mL FGF10; 50 nM ATRA, 3 µM CHIR-99021]. Complete media changes were performed once a week by replacing 100 mL of the culture medium by 100 mL 2x BLO medium (Van Heuve et al., 2025).

### Immunohistochemistry

Immunohistochemistry (IHC) and immunofluorescence staining was performed on formalin-fixed and paraffin-embedded LuOrgs as previously described (Budeus et al., 2024; Ketteler et al., 2020). Following the respective bioreactor runs, some of the LuOrgs were treated with 4% para-formaldehyde/phosphate buffered saline (PBS) for 30 min, and subsequently subjected for paraffin-embedding and sectioning (3–5 µm). Sections were stained with hematoxylin and eosin (HE) and Periodic Acid-Schiff (PAS) staining (to detect carbohydrates, glycogen, mucopolysaccharides, and glycoproteins) (all Carl Roth Karlsruhe, Germany) according to the manufacture’s protocols for histological evaluation. Prior immunofluorescent staining, samples were prepared by using a descending alcohol series and incubation with target retrieval solution (DAKO). Afterward, slides were blocked with a 2% normal goat serum/PBS blocking solution to reduce unspecific interactions, and primary antibodies were incubated overnight at 4 °C. Antigens were detected by fluorescently labeled secondary antibodies. Nuclei were counterstained with Hoechst 33,342. The following antibodies were used. SMA/ACTA2 (B4; #sc-53142) and PDPL (18H5; #sc-59347) antibodies were from Santa Cruz Biotechnology, Inc (Dallas, TX). Acetyl-alpha Tubulin (Lys40) (6-11B-1; # 32–2700) antibody was from ThermoFischer Scientific (Waltham, MA). HOPX (#11419-1-AP), AQP5 (#20334-1-AP), CAV-1 (#16447-1-AP) and TTF1/NKX2-1 (#66034-1-Ig) antibodies were from Proteintech Group, Inc. (Rosemont, IL).

### Bulk and single cell RNA sequencing analysis

Total RNA was isolated and processed as previously described (Sentek et al., 2024; Budeus et al., 2023). RNA concentration and quality were determined with Qubit (Invitrogen, Waltham, MA, USA) and Agilent Bioanalyzer DNA HS (Agilent, Santa Clara, CA USA). Library preparation was performed with Lexogens QuantSeq 3′mRNA-Seq Library Prep Kit FWD and sequenced on a NextSeq500 (Illumina, San Diego, CA, USA). Sequences were trimmed with TrimGalore with standard settings and aligned with hisat2 to hg38 with standard settings. ScRNAseq was performed as previously described (Budeus et al., 2024; Budeus et al., 2025b; Buchholzki et al., 2025). In brief, single cell suspensions were generated by re-suspending LuOrgs in TrypLE (Thermo Fisher Scientific; #12604013) containing 100 unit/mL DNase I followed by a 15-min incubation at 37 °C. Digestion was stopped by adding PBS containing 2%–5% fetal calf serum (FCS), 2 mM EDTA and DNAse I. The cellular solution was passed through a 70 μm cell strainer. Cell viabilities were determined following staining with the cell permeable viability dye Calcein AM in combination with the anthracycline derivative DRAQ7 for nuclei staining of dead cells using the Hemocytometer Adapter of the Rhapsody™HTSingle-Cell Analysis System (BD, Bioscience, Franklin Lakes, NJ); and the viability was always >90%. Single cell suspensions of LuOrgs were then labelled using the BD® Hu Single-Cell Multiplexing Kit (BD Bioscience; #633781) according to the manufacturer’s instructions. Samples were afterwards combined and loaded on one lane on a BD Rhapsody HT Xpress System aiming for 20.000 cells. BD Rhapsody library prep was performed according to the manufacturer’s instructions and sequenced. BDs cwl-runner was used to align and pre-analyze the data. Further analyses were conducted with R packages (Seurat v.4, Enhanced Volcano, scPubr, fgsea, nVennR) as previously described (Budeus et al., 2024; Budeus et al., 2025b; Sentek et al., 2024; Budeus et al., 2023). CytoTRACE (Cellular (Cyto) Trajectory Reconstruction Analysis using gene Counts and Expression) was used to determine the differentiation state of single cells (Gulati et al., 2020).

### Statistical analysis

If not otherwise indicated (n = biological replicates), data were obtained from at least three independent experiments. Data were presented as individual symbols and median values or Interquartile range (IQR). Statistical analysis was performed with R using the R-packages DESeq2 for main DEG calculations. Statistical significance was set at the level of p ≤ 0.05.

## Results

**FIGURE 1:**
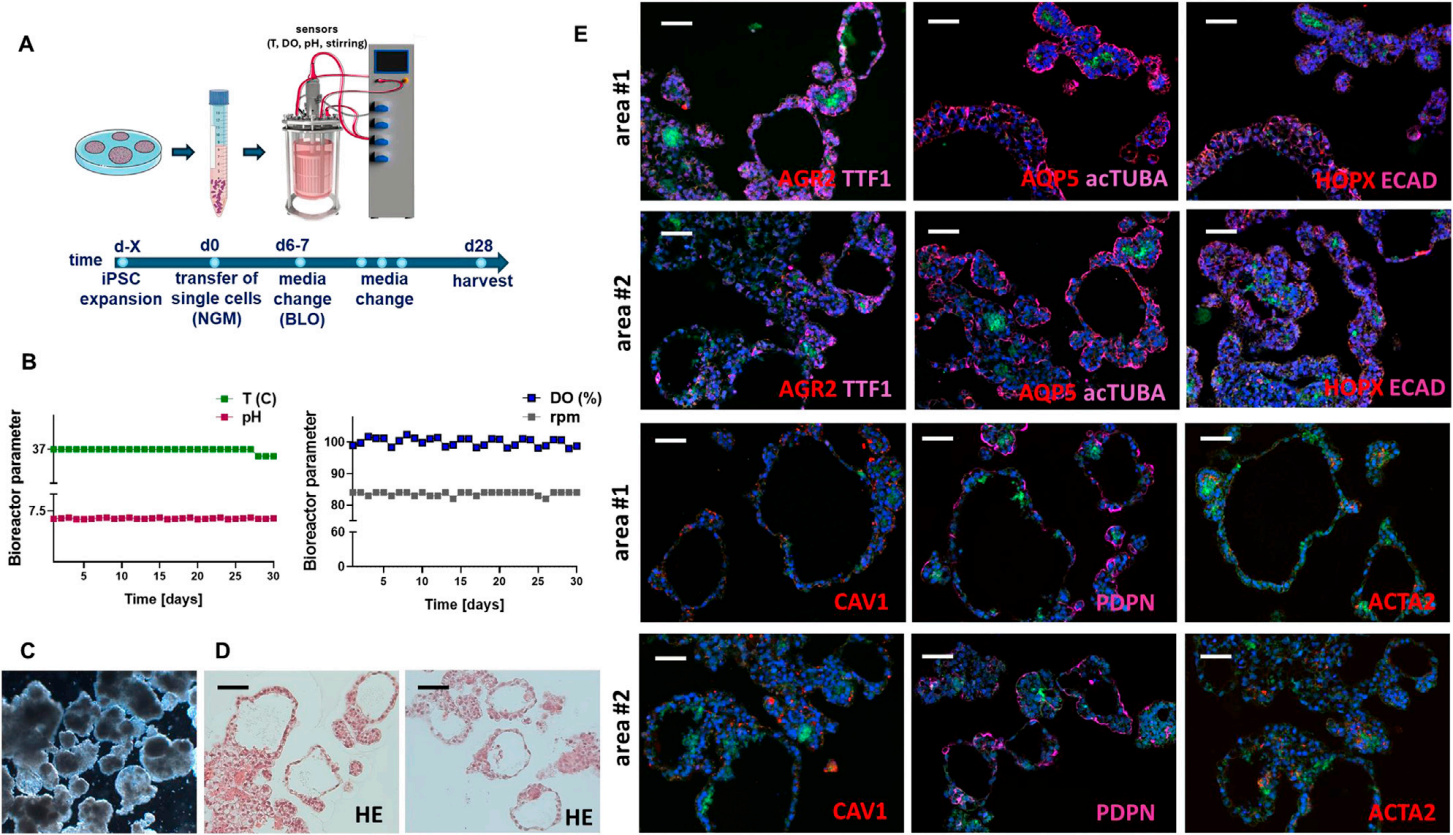
Experimental design and morphological LuOrg characterization **(A)** Scheme for the experimental design including the time line. Expanded iPSCs were harvested using an enzyme-free cell dissociation reagent and then directly transferred to the bioreactor (6.250 cells/mL; 200 mL in total) and cultured for 4–5 days before the normal growth medium (mTeSR1 medium) was replaced with differentiation media (BLO). A regular media change was then performed once a week **(B)** Indicated bioreactor parameters were continuously recorded (DO, dissolved oxygen). **(C)** A representative dark-field image of LuOrgs generated matrix-free from human iPSCs in the Bioreactor at the 28-day time point (end of experiment) using the ×10 objective of an inverted microscope is shown **(D)** Representative hematoxylin and eosin staining (different lung-like regions) of paraffin-embedded sections are shown: 10x; scale bar 100 µm **(E)** Immunofluorescence staining of paraffin-embedded sections of LuOrgs representing different the lung-like regions (area #1, #2) for indicated lung cell type-marker proteins. Nuclei were counterstained using Hoechst 83342. The autofluorescence visible as a muted green indicates the presence of extracellular matrix molecules. Magnification: ×20 objective; scale bar 50 µm. See also Supplementary Figures S1, S2.

Human iPSCs were expanded, dissociated and then directly transferred to the equilibrated bioreactor exhibited with an integrated membrane stirrer (constant agitation with 80 rpm) in NGM (Figure 1A). Dissolved oxygen (DO), pH, temperature and agitation were recorded over time, 28 days in total (Figure 1B). After 4–5 days, a half media change was performed by replacing 100 mL NGM by 100 mL BLO-Medium to induce differentiation (200 mL total culture volume). Complete media changes were performed once a week (d7, d14, d21). Generated LuOrgs were harvested after 28 days of bioreactor cultivation Supplementary Movie 1). Morphological analyses by phase contrast microscopy (Figure 1C; Supplementary Figure S1) and histology (Figure 1D; Supplementary Figure S1) as well as immunofluorescence (Figure 1E; Supplementary Figure S2) showed the efficient generation of lung structures, which seemed to be more of alveolar nature. The structures formed are rather small and indicate the formation of blister-like structures (Figures 1C,D; Supplementary Figure S1) that express the alveolar markers TTF1/NKX2-1, HOPX, AQP5 and PDPN, while cells that are positive for the mesenchymal marker ACTA2 are only found sporadically.

**FIGURE 2:**
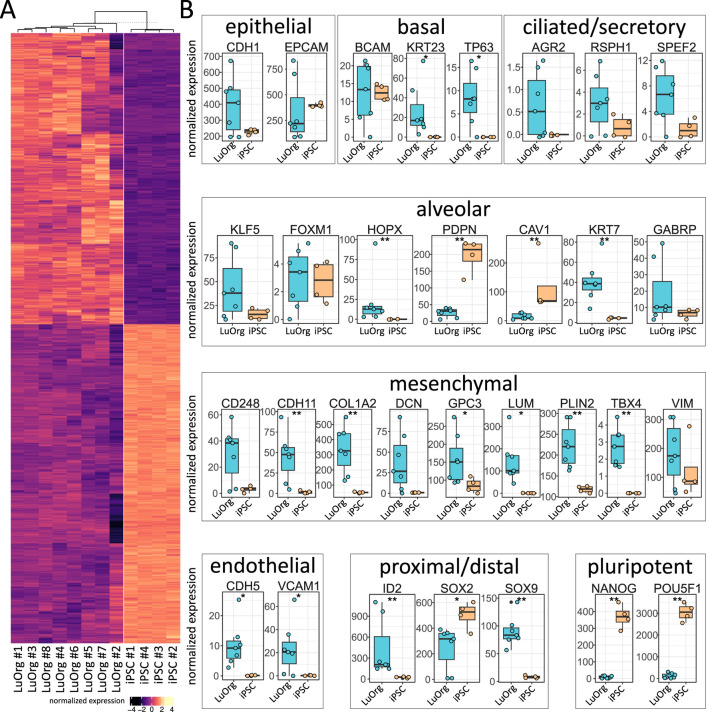
Molecular analysis of generated LuOrgs (bulk RNAseq) **(A)** Heatmap of top 2500 normalized expressed genes of donor cells (d0, iPSCs, four samples), and bioreactor-generated LuOrgs (d28, 8 samples). Biological replicates as indicated. Heatmap is row-normalized to show differences between the samples and not the genes **(B)** Normalized expression of marker genes in iPSCs and generated LuOrgs for epithelial, basal, ciliated/secretory, alveolar, AECI, fibroblastic, endothelial and pluripotent cells are shown as box plots. Mann-Whitney *U* test. * = 0.05, ** = 0.01. See also Supplementary Figure S3.

We performed gene expression analyses of respective LuOrgs compared to donor cells (iPSCs) to confirm the differentiation of bioreactor-generated LuOrgs directly from iPSCs (Figure 2). A heatmap of all LuOrg and iPSC samples including hierarchical clustering of the top 2500 expressed genes shows a clear separation between both (Figure 2A). Gene set enrichment analysis (GSEA) according to the HE_LIM_SUN (He et al., 2022) lung cell gene set confirmed the successful generation of the most relevant lung cell types while immune subsets were lacking (Supplementary Figure S3). Comparative analyses of normalized RNA counts further highlight significantly upregulated expression levels of typical lung cells including basal and bronchioalveolar stem cell (BASC) marker (TP63, KRT23), ciliated/secretory (AGR2, RSPH1, SPEF2) and particularly upregulated expression levels of the alveolar lineage maker KLF5, SOX9, ID2 and KLF5 together with the alveolar epithelial cell type 1 (AECI) (HOPX), and the AECII (KRT7, GABRP) marker genes (Figure 2B). Significant upregulated expression levels of mesodermal genes include CDH11, COL1A2, GPC3, LUM, TBX4 and PLIN2. Also, a significant upregulation of endothelial genes was detected. As expected, significant declines in NANOG and POU5F1/OCT4 expression were determined. Quantitative Real Time RT-PCR confirmed increased expression levels of the distal marker gene ID2 compared to the proximal/bronchial marker gene FOXJ1 (Supplementary Figure S4).

**FIGURE 3:**
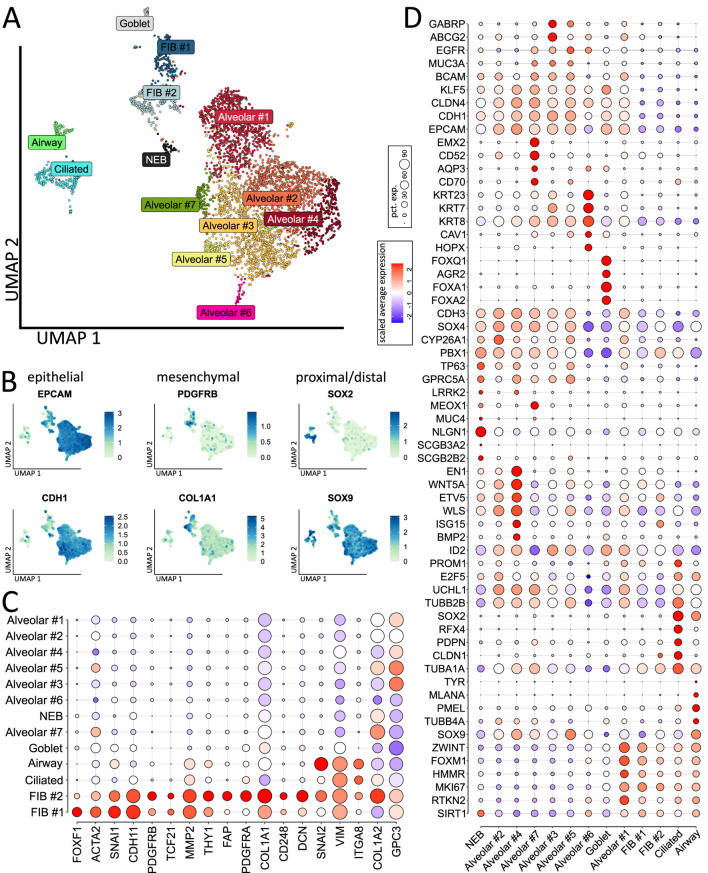
Composition of bioreactor-generated LuOrgs (single cell RNAseq) **(A)** Uniform Manifold Approximation and Projection for Dimension Reduction (UMAP) of all single cells colored by identified clusters. Cell-type labels for each cluster are based on expression of canonical cell-type markers displayed in the following plots **(B)** Expression map of representative epithelial (EPCAM, CDH1), mesenchymal (PDGFRB, COL1A1) marker genes, or SOX2 and SOX9 for proximal-distal patterning in all clusters **(C)** Dot plot of indicated mesenchymal marker genes **(D)** Dot plot of epithelial marker genes. Size of the dots indicate the percentage of cells in which this gene was found, the color indicates the normalized value of the expression.

We next performed single cell RNA-seq of respective LuOrgs to characterize the bioreactor-generated lung cell phenotypes in more detail and to verify the presence of different epithelial and mesodermal lung cell subsets (Figure 3; Supplementary Figure S5). Single cell clustering revealed the presence of 13 different main cellular subsets (Figure 3A). A distribution of epithelial genes EpCAM/CD326 and ECAD/CDH1 compared to mesodermal genes PDGFRB and COL1A1 clearly indicated the predominant formation of epithelial lung cells (Figure 3B). Distal epithelial cell types, indicated by the prominent SOX9 expressions, were significantly more prevalent compared to SOX2-positive proximal epithelial cells. Among the mesodermal cell types two clusters were identified characterized by general expression of mesenchymal gene expressions like CDH11, VIM, and CDH11 and PDGFRB (Figure 3C; Supplementary Figure S3). The designated FIB #1 cluster was characterized by the expression of FOXF1, ACTA2, PRRX1 and HOXA10 and thus might represent alveolar (interstitial) fibroblasts. FIB #2 fibroblasts were characterized by increased expression levels more reactive ECM-remodeling related genes, e.g., DCN and Endosialin/CD248 together with FAP and COL1A2 and were designated as adventitial fibroblasts including myofibroblasts. Secretory cells and particular goblet cells were identified by the expression of FOXA1/A2, ARG2, and FOXQ1 (Figure 3D; Supplementary Figure S5). Proximal cells, particularly ciliated cells, were identified by the expression of acetylated tubulin (TUBA1A) together with TUBB2B, RFX4, CLDN11 and SOX2, close to a cluster of cells (designated as airway) expressing PMEL, MLANA, TUBB4A and FOXD3, the first of which are known to be expressed by LAM-like cells LAM-like cells (Guo et al., 2020). Based on the expression of NLGN1, NELL1, CNKSR2, and ZC3H12 -genes indicative of the capacity to store and release neurotransmitters-the corresponding small cluster might contain pulmonary neuroepithelial body (NEB) cells. Seven alveolar clusters were further identified. Cluster alveolar #1 consisted of proliferating alveolar progenitor cells (APC) expressing MKI67, HMMR together with alveolar marker genes ID2, FOXM1, ZWINT and RTKN2. The cluster designated alveolar #2 is located directly next to it, but does not actually show any clear marker genes. Among the top expressed genes here, although being low compared to other cluster-specific gene, CYP26A1, PROM1 and FGF9 became apparent. This suggests early-stage epithelial cells being prone for epithelial budding lung branching. The alveolar #3 cluster was designated as AECII-prone or AECII progenitor cells based on the expression of GABRP, KRT7, ABCG2, AHNAK and CD9. The cells of alveolar cluster #4 were characterized by the expression of ETV5, BMP2, WNT4A, and WLS, expression patterns that indicate an AECII phenotype and could also suggest the presence of rare AECII stem cells (Nabhan et al., 2018). Cells of the alveolar #5 cluster, close to the AECII progenitor cells (cluster #3) seemed to be highly glycolytic active as indicated by the expression of SLC1A6, SLC16A3, PDK1, PFKFB4 and HK2. These cells were designated as AECII cells differentiating into AECI cells, as this process is known to be energetically demanding (Wang et al., 2023). In the immediate vicinity, small cluster #6 is found; it is characterized by the expression of the AECI markers HOPX and CAV1, the AECII marker KRT7, and the para-/supra-basal marker KRT23. This points to AECI cells—or AECII cells differentiating into AECI cells -with the differentiation into AECI appearing to be significantly more advanced in this instance. Finally, within the alveolar cluster region, alveolar cluster #7 were characterized by the expression of the AECII marker AQP3 and the transitional stem cell marker KRT8, together with TUB1A1 and EMX2, the latter one being an essential transcription factor for cilia development. Thus, these cells could represent common multipotent progenitors for bronchiolar and alveolar progenitors, and thus represent bronchioalveolar stem cells (BASCs).

**FIGURE 4:**
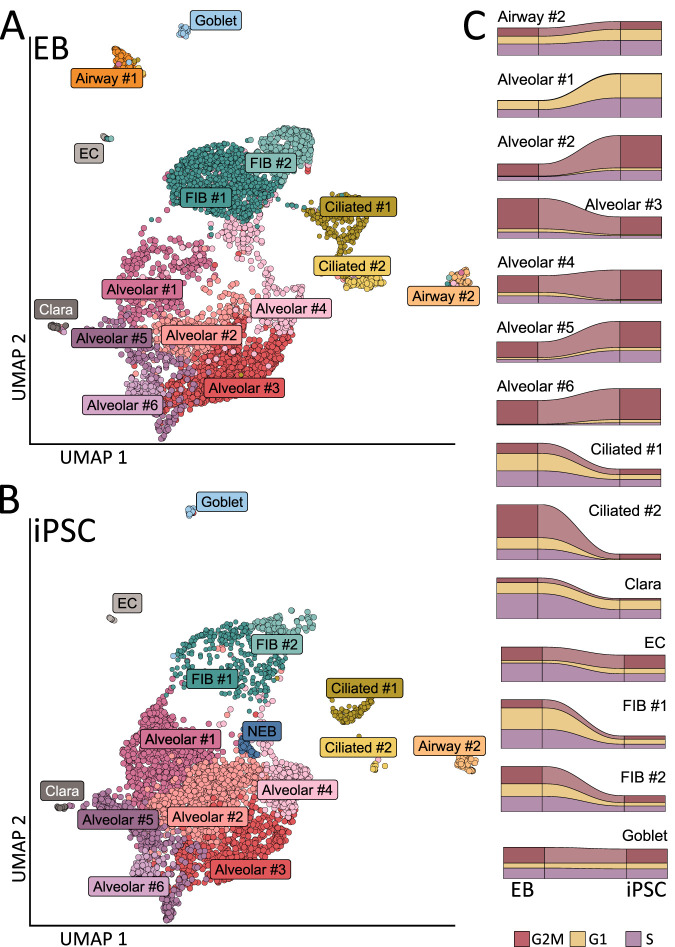
Comparison of the bioreactor differentiation of LuOrgs generated from embryoid bodies versus directly from iPSCs. The scRNAseq dataset from bioreactor-generated LuOrgs directly from iPSCs (Figure 3) were integrated with previously published scRNAseq datasets from bioreactor-generated LuOrgs derived from embryoid bodies (EB) (Budeus et al., 2025b) **(A, B)** Both single cell datasets were re-clustered together and both UMAP of all single cells colored by identified clusters are depicted (A, EB; B, iPSC). See also Supplementary Figures S6, S7. **(C)** Cell cycle classification per cluster in bioreactor-generated LuOrgs either of iPSCs or EBs as starting point.

**FIGURE 5:**
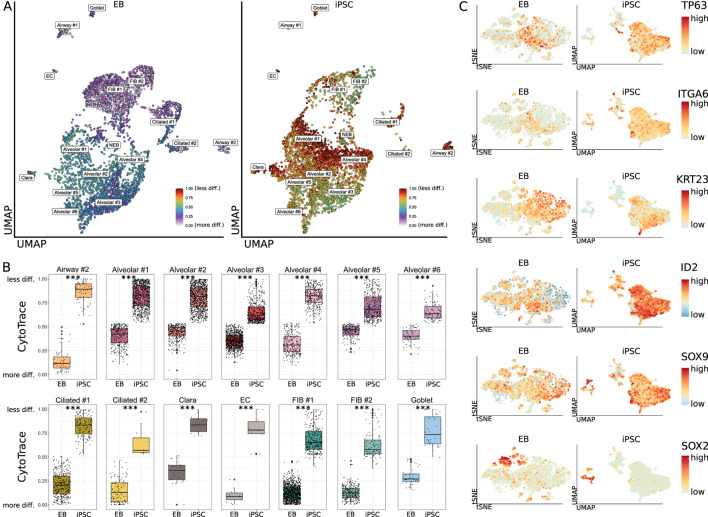
Differentiation state of EB and iPSC BR cells **(A)** UMAP of CytoTrace score for all single cells in iPSC and EB experiment in integrated orientation. Please note that the colors on top (redish) identify less differentiated cells **(B)** Boxplot of CytoTrace scores for each individual cluster of the integrated cells comparing EB and iPSC origin. Please note that high values means less differentiated **(C)** Feature plots showing the gene expression of certain genes important for differentiation in EB and iPSC cells.

In short, direct culture of iPSCs followed by differentiation in the bioreactor predominantly yielded LuOrgs, which are mostly alveolar in nature. In contrast, the culture of pre-differentiated iPSCs in the form of EBs in the bioreactor generated LuOrgs that very well exhibited bronchiolar and alveolar lung structures (Budeus et al., 2025b). Therefore, we examined the difference between the two Bioreactor cultures (Figure 4A; Supplementary Figure S6). For this purpose, the single-cell datasets from both culture conditions were merged and re-clustered (Figure 4A; Supplementary Figure S7). This revealed that certain clusters emerged identically in both approaches, namely, the goblet, clara and endothelial cell clusters as well as cells in the airway #2 cluster that expressed LAM-like genes. The cluster designated as airway #1 of immune conferring cells (expressing IDO1, IL1B, C3 and CXCL8) (Budeus et al., 2025b) was only found in the EB-differentiated bioreactor runs, while the NEB cluster was only found in the bioreactor runs -as shown here-that directly received iPSCs as starting cells. Fibroblast, ciliated and alveolar cell clusters were also present, but with different cell numbers. Regarding the cell cycle phases predominating in both clusters, no major differences could be observed between the EB bioreactor variant and the one where iPSCs from starting material were used (Figure 4B). However, these differences do indicate changes in cellular composition, namely, fewer ciliated and mesodermal cells in the direct iPSC bioreactor cultivation. We then used a publicly available computational tool to assess the degree of differentiation of our bioreactor-generated lung organoids. To this end, the differentiation state of cells was predicted from single-cell RNA sequencing data using the CytoTRACE (Gulati et al., 2020) method (Figure 5). It was found that the LuOrg cells differentiated directly from iPSCs in the bioreactor appear overall less differentiated (Figure 5A); in particular, the differentiation stages within individual clusters were significantly lower compared to the corresponding cell clusters in LuOrgs differentiated from EBs in the bioreactor (Figure 5B). Consistent with this, it has been shown that lung stem cell markers (TP63, ITGA6, KRT23) in these LuOrgs are more widely distributed across the entire cell population, rather than being localized in specific clusters as is the case with LuOrgs differentiated from EBs (Figure 5C). A similar pattern is observed for genes associated with the distal-proximal axis (ID2, SOX9, SOX2). It was shown (once again) that distal markers predominate in the cells of lung organoids generated from iPSCs in the bioreactor.

**FIGURE 6:**
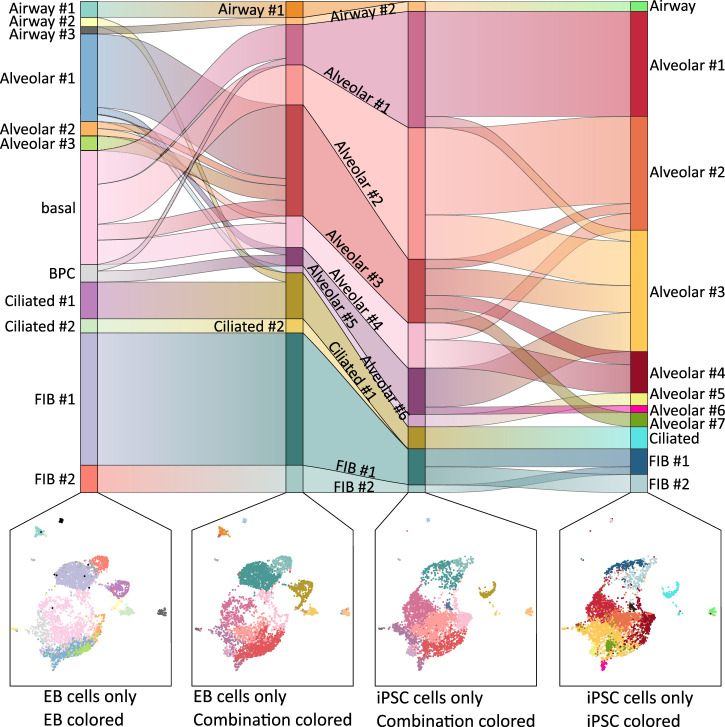
Association and abundance of clusters between EB-based, iPSC-based and combined LuOrg cells. Cluster sizes of the bioreactor-generated LuOrgs comparing EBs as starting material (Budeus et al., 2025b). versus iPSCs. On the left are the cluster names of the previous bioreactor runs (Budeus et al., 2025b), when LuOrgs were differentiated starting from EBs. On the right are the cluster names of the bioreactor runs shown here in Figure 1, when LuOrgs were differentiated starting from iPSCs, and in the middle are the common newly clustered names of both approaches as well as the quantity ratios. Alluvial plot between the two bioreactor samples to show the consistency of clusters. Each of the four colunms shows the abundance of cells in each cluster. To further visualize the associated cells, we show below each column a UMAP with the cells in the column colored accordingly. The first column shows the EB-based LuOrg cells with the identified clusters, the second column shows the same cells, but with the cluster identification of the combined dataset, the third column shows the iPSC-based LuOrg cells, again with the cluster identification of the combined dataset, and the fourth column shows the iPSC-based LuOrg cells, with the cluster identification based only on this sample. The connections between the first and second column as well as the third and fourth column show the flow between the different cluster identifications of the identical cells. The connection between the second and third column show the shift in abundance of certain clusters between EB-based and iPSC-based LuOrgs. Most of the cluster associations are consistent between the LuOrgs from EB vs. iPSC, but the abundance differs. Only cluster and connections with at least 50 cells are shown. See also Supplementary Figure S8.

Next, both runs were examined in more detail to identify differences in the individual clusters, compared to the initial (as shown in Figure 3A) and previous (Supplementary Figure S6; Budeus et al., 2025b) (Figure 6; Supplementary Figure S8). Bioreactor runs initiated from previously pre-differentiated iPSCs in the form of EB (Budeus et al., 2025b) are referred to as EB cultures, while bioreactor runs initiated directly from iPSCs as presented here are referred to as iPSC cultures. The cells of the ciliated #1 cluster, consisting of immature, developing ciliated epithelial cells, are found in both bioreactor runs, but in significantly higher numbers in the EB culture variant. In contrast, the more mature ciliated cells (ciliated #2) are only found sporadically in the EB runs (Figures 4A,B, 6). The cells of the EB airway #2 cluster are also found in the joint ciliated #1 cluster, i.e., luminal progenitor cells designated as supra-basal cells and colloquially referred to as “lung umbrella cells”. As the joint ciliated #1 fully comprises the iPSC ciliated cluster, this also reflects the fact that the ciliated cells of the iPSC bioreactor are still rather immature here.

It became evident that mesodermal cells were significantly less prevalent upon iPSC cultivation. The FIB#1 cluster generally showed cells expressing mesenchymal genes (Supplementary Figure S7), which represented mesenchymal stem cell-like cells or, more crudely, fibroblastic precursors including alveolar (interstitial) fibroblasts. The FIB#2 cluster contained cells expressing genes that indicated a more reactive phenotype (ACTA2, CD248, DCN, POSTN, SERPINE1) and were accordingly classified as FIB#2 (intestinal fibroblasts including myofibroblasts) (Supplementary Figure S7). The EB FIB #1 cluster representing alveolar (interstitial) fibroblasts and the EB FIB #2 cluster representing adventitial fibroblasts (including myofibroblasts) were also reflected in the joint clustering (Figures 4A, 6). Both iPSC FIB clusters are found together in the FIB#1 cluster. It turned out that the cluster designated iPSC FIB#1, representing alveolar (interstitial) fibroblasts, also contains cells that express more RGS5, PDPN, and FLT1, which is indicative for pericytes were found within EB cluster FIB #2 and so in the FIB#2 cluster of the joint clustering, together with lower levels of proliferative genes (PCNA, KI67 and HMMR, Supplementary Figure S8). The iPSC FIB#2 cluster, which expressed more reactive ECM-remodeling related genes compared to the iPSC FIB#1 cluster, is found together in the FIB#1 cluster. These cells seemed to bear a less reactive phenotype compared to the adventitial cells of the EB FIB#2 cluster.

The greatest differences in the overall clustering, however, were evident with regard to the alveolar cells. In EB we could separate four different alveolar clusters (basal, including BASC) and alveolar #1 (AECII-differentiating into AECI cells), alveolar #2 (AECI/II-differentiating BASC), and alveolar #3 AECI cells). In the joint clustering, these became the alveolar clusters #1-#6 (Figures 4A, 6). The cells of the EB basal cluster were distributed across alveolar clusters #1-#4, indicating that a certain basal cell nature predominates in the shared clustering and thus also in the iPSC alveolar clusters, confirming less differentiation. In the shared clustering, proliferating alveolar progenitors (APCs) are evident in alveolar cluster #1, expressing not only MKI67 and HMMR but also BCAM, and KRT23, in particular FOXM1, KRT7, and GABRP (Supplementary Figure S8). Correspondingly, the cells of the iPSC alveolar cluster #1 are also found in this cluster. Approximately one-third of the cells from the EB basal cell cluster contribute to the shared alveolar cluster #1, which further specified these cells as basal cells that are already differentiating towards APCs. Approximately another third of the EB basal cell clusters were found in common alveolar cluster #2, which on the other (iPSC) side consists of cells from clusters alveolar #2 and #3. Common alveolar #2 did not present a clear picture. As described above, it presumably contained early-stage epithelial cells prone for epithelial budding lung branching, thus also rather immature, basal-like cells.

Common alveolar #3 cluster showed high expression levels of KRT4, KRT17, and KRT23, along with the AEC1 marker genes HOPX, GPRC5A, and CYP26B1, meaning these were AT1 cells or stem cells differentiating into AT1. The expression of KRT8 also suggests a transitional stem cell state that precedes the regeneration of AEC1 cells (Strunz et al., 2020) (Figure 3A; Supplementary Figure S5; Supplementary Figure S8). In line with this, the cells identified as AEC1 in the EB alveolar #1 cluster contribute significantly to this common cluster, as do some cells from the EB basal cell cluster. This means that on the EB side, among the basal cells, there are already specific AT1 precursors, most likely BASC cells that differentiate into AT1, or alveolar intermediate basaloid cells (Angeles-Lopez et al., 2025), indicated by KRT17 expression. From the iPSC side, cells from several alveolar clusters contribute to this common cluster alveolar #3 (Figures 4A, 6A). The common alveolar #4 cluster was contributed by basal cells from the EB side that express several mesenchymal genes (VIM, COL1A1, TWIST1, POSTN), suggesting a subset of mesenchymal progenitors, but also by cells from the EB alveolar cluster #2, which represent AECI/II lineage-prone cells or, more specifically, AECI/II-differentiating bronchioalveolar stem cells (Budeus et al., 2025b). Conversely, cells from alveolar clusters #2 and #4, and thus more alveolar stem cells with AEC2 characteristics, contributed to the cluster from the iPSC side. The common alveolar #5 cluster consisted, from the EB side, of cells from the EB alveolar #1 cluster, namely, AECII cells, including AECII differentiating into AECII and bronchial progenitor cells (BPCs) expressing acetylated tubulin (TUBA1A) together with TUBB2A/B (Supplementary Figure S8). The simultaneous expression of the alveolar markers GABRP, AQP11, and KRT7 within this cluster might indicate the bronchioalveolar stem cell nature of these cells, which are differentiating towards bronchiolar cells. Finally, the small alveolar #6 cluster consisted of cells that are highly glycolytically active (ENO2, HK2, PFKFB4). From the EB side, these are AT2 cells differentiating into AT1 cells. This also fits from the iPSC side. Here a small part of the cluster #1 cells, which initially consisted of alveolar epithelial cells type 2 (AECII cells) including AECII-differentiating into AECII cells, based on the expression of GABRP, KRT7, MUC15 and LAMP3 together with GPRC5A and HOPX (Budeus et al., 2025b).

## Discussion

iPSC-LuOrgs have proven invaluable for the modeling of lung diseases. Large-scale bioreactor production of these lung structures would even facilitate toxicity testing and potentially also clinical applications. The aim of this study was to investigate whether LuOrgs can be efficiently generated in a bioreactor using directly iPSCs as starting cells. It was hypothesized that the introduced iPSCs (in NGM/mTeSR1 medium) would initially form EBs or at least EB-like structures, as these cannot adhere to a coated plastic surface. While the formation of cellular aggregates of varying sizes was observed in NGM 4–5 days after initial bioreactor cultivation (not shown), the aggregates did not appear to be EBs of sufficient quality, as efficient differentiation into lung tissue with bronchial structures alongside the alveolar ones failed to occur. These cellular aggregates could also resemble inter-EB agglomerations, which are known to inhibit cell growth and differentiation in stirred or high-cell-density static cultures applications (Dang et al., 2004). But as epithelial-mesenchymal interactions are of particular importance for normal lung development and homeostasis, a multi-germ-layer involvement for LuOrg generation is required (Mitchell et al., 2023; Tamai et al., 2022), a fact that could be addressed by using EBs as starting point. This could explain why predominantly epithelial structures of an alveolar nature were generated in this approach, and only a few bronchial and mesenchymal cell types could be found. Thus, the differentiation of iPSCs in EBs seems to be a necessary prerequisite for the efficient generation of iPSC-LuOrgs -at least in our bioreactor set up-, which then exhibit very good mesenchymal and alveolar cell types and structures, as well as lung structures of a bronchial nature (Budeus et al., 2025b; van Heuvel et al., 2025). It could, of course, also be that the cultivation process in the bioreactor simply requires more time when iPSCs were introduced. In contrast, however, the proportion of airway cells, especially ciliated cells, was low and, moreover, mostly in an immature stage. We will address this finding in future studies through adapted conditions such as the semi-solidification of the medium and/or the timed addition of growth factors, so we can achieve a direct generation of LuOrgs in the bioreactor without having to rely on steps outside the system beforehand. In addition to the possibilities of forcing the formation of EBs in bioreactor in terms of pre-differentiation, the latter, the timed addition of inhibitors and growth factors, could also be used to influence the development of favorable LuOrg phenotypes. For example, FGF10 is known to foster generation of alveolar- and airway-like cell types from lung progenitor cells whereas ATRA, FGF7, and CHIR directs differentiation towards of bud tip organoids containing AECII, and club and goblet cells (Miller et al., 2020; Alsobaie et al., 2023; Jacob et al., 2017). Variations in the concentration and/or timing of these factors in the bioreactor could lead to improvements in the cellular composition and maturation of the LuOrgs, although further optimization regarding more accurately recapitulating the lung architecture would then be necessary (Heinzelmann et al., 2024).

Generally, iPSC-derived alveolar organoids can efficiently be generated based on established protocols, when (human) iPSCs were sequentially induced to definitive endoderm, anterior foregut endoderm, and (NKX2-1- positive) lung progenitor cells that are further differentiated into distal alveolospheres (Gandikota et al., 2024; Jacob et al., 2019). In this process, following differentiation into definitive endoderm, cells are seeded onto plates coated with growth factor-reduced (GFR) Matrigel and subsequently differentiated further. Sorting NKX2.1-positive cells via flow cytometry prior to subsequent cell differentiation further improved the cellular composition and the differentiation stage of the resulting lung organoids (Hein et al., 2022; Castaneda et al., 2023). In addition, air-liquid interface (ALI) cultures, serve as complex *in vitro* models of the airway and alveolar epithelium facilitating improved cell differentiation. Together with the simultaneously optimization of the morphological and histological properties of epithelial cells, these cultures have proven highly advantageous for respiratory modeling studies and developing drugs for lung diseases (Ito, 2026; Silva et al., 2023). Primary lung epithelial cells with stem cell properties -isolated predominantly from patient samples- or alveolar and bronchial cell lines are used for these cultures. A direct comparison of this methodology with corresponding lung organoids demonstrated comparability between the different models, at least regarding the presence of relevant cell types, assessed viral replication kinetics, and antiviral cytokine responses (Ekanger et al., 2025). However, patient material is available only to a limited extent, and not every laboratory has access to it. Furthermore, there is considerable donor variability, driven by factors such as age, treatment, and the quality of the subsequent cell isolation. These limitations can be circumvented by using iPSCs as starting cells, which in turn would also circumvent disadvantages regarding immunogenicity (in the context of cell or organ replacement therapy) as well as potential ethical concerns (Klein, 2018). To reflect patient-specific disease states, more complex, possibly genetic, modifications would then be necessary, which would of course not be required with patient-derived organoids. In contrast, iPSC-derived alveolar organoids allow to model diseases that cannot be easily tested in humans (Kim et al., 2024), and developmental stages can also be modeled in iPSC organoids overall (Heinzelmann et al., 2024). Finally, the 3D structure of lung organoids—compared to 2D cultivation methods—is naturally a decisive advantage that makes the entire system more *in vivo*-like. This, combined with optimal nutrient supply in the bioreactor (since static conditions are avoided), could enhance the differentiation of human iPSCs (Alsobaie et al., 2023). As previously mentioned, the latter aspect requires further optimization for the current setup.

However, it is also crucial to identify new regulators of alveolar organoid formation in order to expand our knowledge at the molecular level in relation to the specific cell type, which could ultimately lead to a more targeted approach to lung repair in lung diseases (Purev et al., 2024). And scRNAseq was used to gain the knowledge of precise and differential information in a cell-type dependent manner (Wang et al., 2018). By comparing the two bioreactor cultivation variants and thus by integration of the different single-cell datasets, new genes could be identified that are important for the lungs as a whole, but especially for typing the different lung cell populations. These genes could therefore be used as marker genes in the future, or serve as a molecular basis for further functional investigations. For example, energy production via upregulated glycolysis is an important prerequisite of alveolar stem cells to initiate alveolar (re-)generation as the process of AECII differentiating into AECI is an energetically costly process (Wang et al., 2023). Genes that were found to be highly expressed here were ENO2, HK2, PFKFB4, and also SLC16A3, a high affinity lactate transporter with physiologically relevant affinity for pyruvate and the glutamate transporter SLC1A6. On the other hand, it is still unclear how stem cells are selected from bulk AECII, and therefore how they are maintained (Nabhan et al., 2018). Other markers for AECII included KRT7 and AHNAK, with the loss of the latter already associated with, type II pneumocytes hyperplasia by suppressing proliferation by activating the TGFβ signaling pathway (Park et al., 2018). Since BMP signaling is active in AECII (Chung et al., 2018), increased gene expression of the family member BMP2 could be identified here. In alveolar cells, and especially AECI cells, KRT15 and KRT39 appear as further potential marker genes, as does CYP26B1, for which loss has already been shown to be associated with fewer AEC1 cells (Daniel et al., 2020). Concerning the mechanism it was suggested that CYP26B1 may reduce retinoic acid signaling thereby promoting proper distal epithelial differentiation in a paracrine manner (Daniel et al., 2020). FOXM1 in contrast, a known and moreover crucial transcription regulator of alveolar epithelial cells, seemed to be a more general lung cell marker, as expression of FOXM1 was also detected in cells of ciliated and fibroblastic clusters, which is in line with the role of FOXM1 during embryogenesis and the development of lung disease (Chen et al., 2024; Li et al., 2019b). For larger airway epithelial cell types PROM1, together with FGF9 was found to be expressed in cells designated as early-stage epithelial, as Fgf9 was found to be expressed in lung epithelium during the first half of the pseudo-glandular stage of development (Yin and Ornitz, 2020). PROM1, a transmembrane protein widely used as stem cell marker, is known to be expressed at distinct levels along the proximal-distal axis of the airways and becomes then becomes restricted to multi-ciliated cells, where it modulates cilia length and ciliary beating frequency in a NOTCH-dependent manner (Serra et al., 2022). Among the mesodermal genes, CRISPLD2 and RGS5 came up in one of the fibroblast clusters. CRISPLD2 was identified as an important lung function gene which fibroblastic expression is important for fetal lung development and later on in adult lung homeostasis by limiting production of inflammatory cytokines (Zhang et al., 2016; Zhang et al., 2015; Kachroo et al., 2019). RGS5, a marker for mural cells particularly pericytes was shown to be involved in vascular pro-inflammatory and pro-fibrotic phenotypes (Lu et al., 2023; Liu et al., 2021). PRRX1 is another gene, which was found to be highly expressed in interstitial fibroblasts. PRRX1 is known as master transcription factor of stromal fibroblasts for myofibroblastic lineage progression (Lee et al., 2022), and expression of these marker gene could be used for the identification of activated, CAF-like fibroblast subsets prone to proliferate and participate in fibrotic processes (Marchal-Duval et al., 2023). For each cell population generated, new potential marker genes were identified that contribute to the understanding of lung homeostasis and whose changes in disease provide new starting points, even if network analyzes to identify the involved pathways have not yet been carried out here.

In summary, it can be stated that LuOrg generated in the bioreactor starting from iPSCs were more of alveolar nature. For the formation of bronchial structures alongside, as well as an overall more matured state of the LuOrgs, further protocol optimization is required. Nevertheless, the investigations carried out here also provide valuable information about cell type-specific marker genes depending on their condition.

## Data Availability

The single cell RNA-seq data have been deposited at Gene Expression Omnibus (GEO) and are publicly available as of the date of publication (accession number: GSE301233 (20), GSE275539 (18) and GSE337365). Any additional information required to reanalyze the data reported in this paper is available from the corresponding author upon request.
