# Supplementary material for: Generation of alveolar organoids from human induced pluripotent stem cells using a stirred-tank bioreactor
Source: Front Bioeng Biotechnol. 2026 Aug 5;14:1858660. doi: 10.3389/fbioe.2026.1858660 (PMC13485884; doi:10.3389/fbioe.2026.1858660)
Supplement: Supplementary file 1 [file DataSheet1.pdf]

Supplementary Material

Generation of alveolar organoids from human induced pluripotent stem cells using a stirred-tank bioreactor

Bettina Budeus[1], Lea Cremer[1], Zehra Fatma Sevindik[1], Diana Klein[1],*

1Institute for Cell Biology (Cancer Research), Medical Faculty, University of Duisburg-Essen, Essen, Germany

Data availability

The bulk and single cell RNA-seq data have been deposited at Gene Expression Omnibus (GEO) and are publicly available as of the date of publication (accession number: GSE301233, GSE275539 and GSE337365). Any additional information required to reanalyze the data reported in this paper is available from the corresponding author upon request.

Supplemental Movies

Supplemental Movie 1:

Video recording of the formed lung organoids in the bioreactor at the end of cultivation (day 28). The membrane stirrer can be seen in the upper part of the culture vessel. The vessel is illuminated from below with a cell phone flashlight.

Supplemental Figure S1

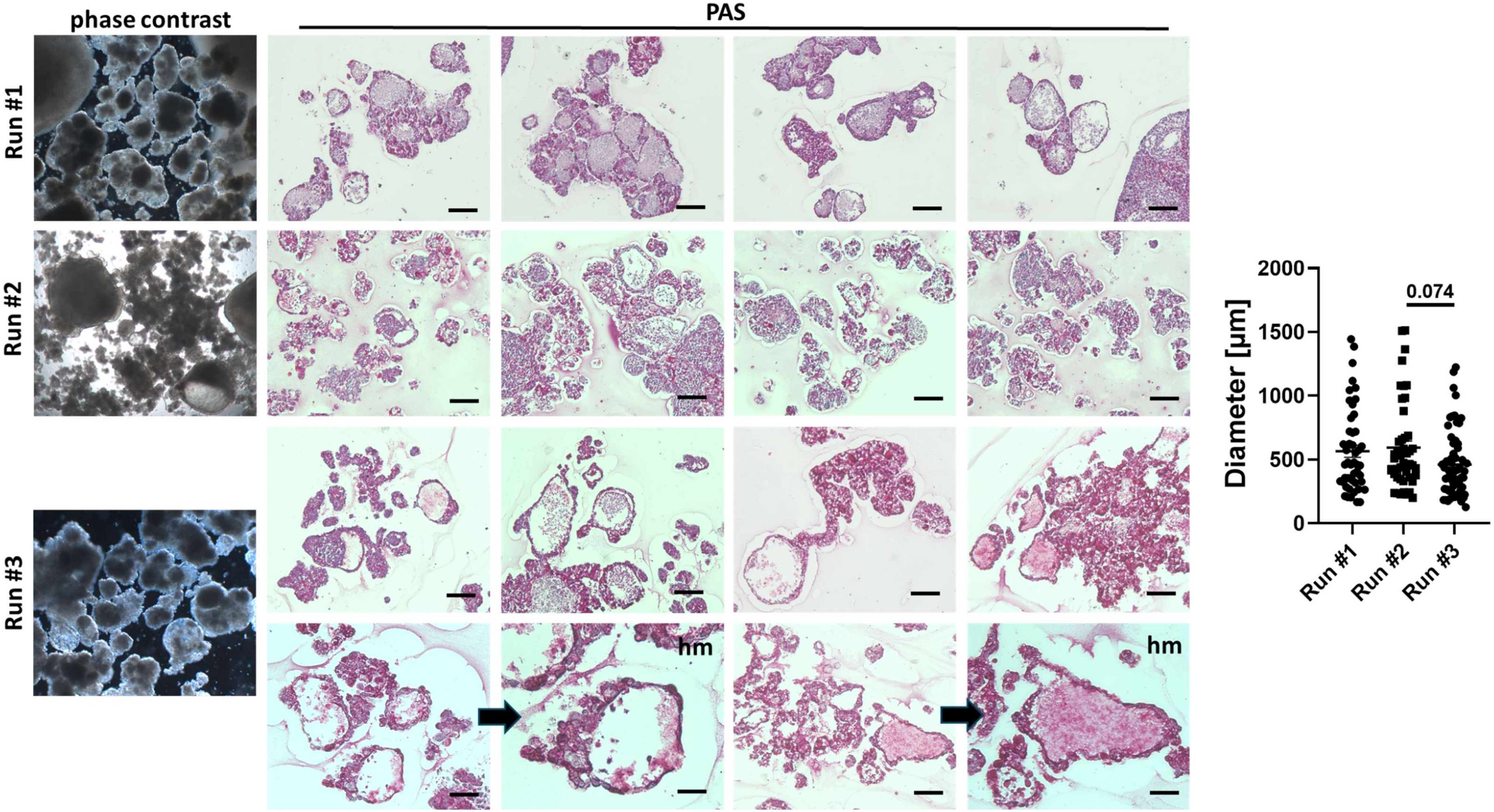

Morphological characterization of LuOrgs generated directly from iPSCs using the bioreactor. Representative phase contrast photographs of three (out of five) different bioreactor runs (Run #1, #2, #) are shown, taken with the 10x objective of an inverted microscope. PAS (Periodic Acid Schiff) staining was performed afterwards of respective paraffin-embedded sections. Different LuOrg structures within the same section per bioreactor run are

exemplarily shown. Magnification: 10x; scale bar 100µm. Higher magnification (hm) images were included to highlight the pink-magenta stained mucus in alveolar lumen. Magnification: 20x; scale bar 50µm. To estimate the size, the diameter of the generated structures was determined on histological sections (as shown) using ImageJ (https://imagej.net/ij/).

Supplemental Figure S2

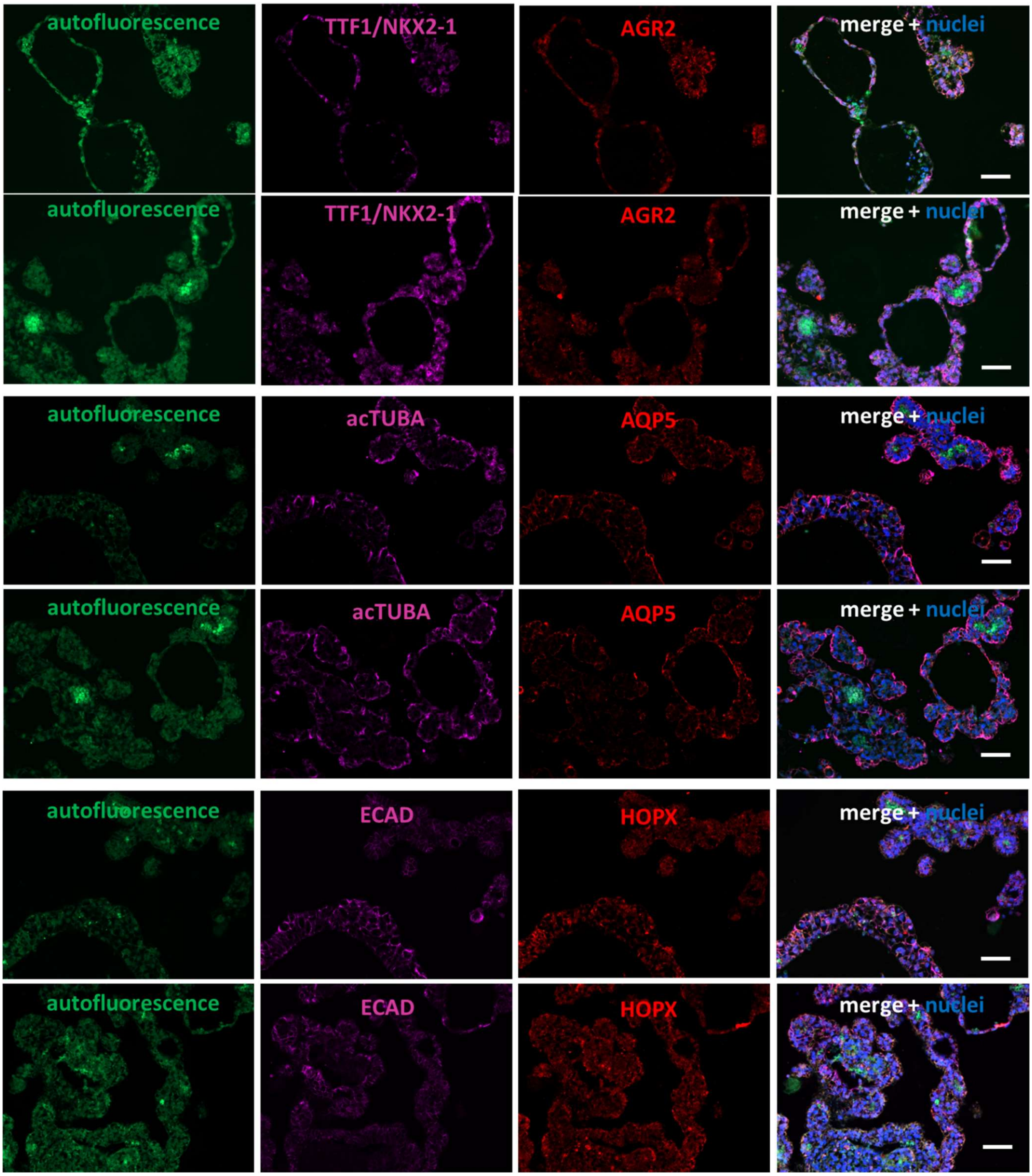

Individual fluorescence channel recordings of the immunofluorescence images shown in Figure 1E. Immunofluorescence staining of paraffin-embedded sections of LuOrgs representing different the lung-like regions (area #1, #2) for indicated lung cell type-marker proteins. Nuclei were counterstained using Hoechst 83342. Autofluorescence properties usually based on extracellular matrix proteins are shown in green. Magnification: 20x objective; scale bar 50µm.

Supplemental Figure S3

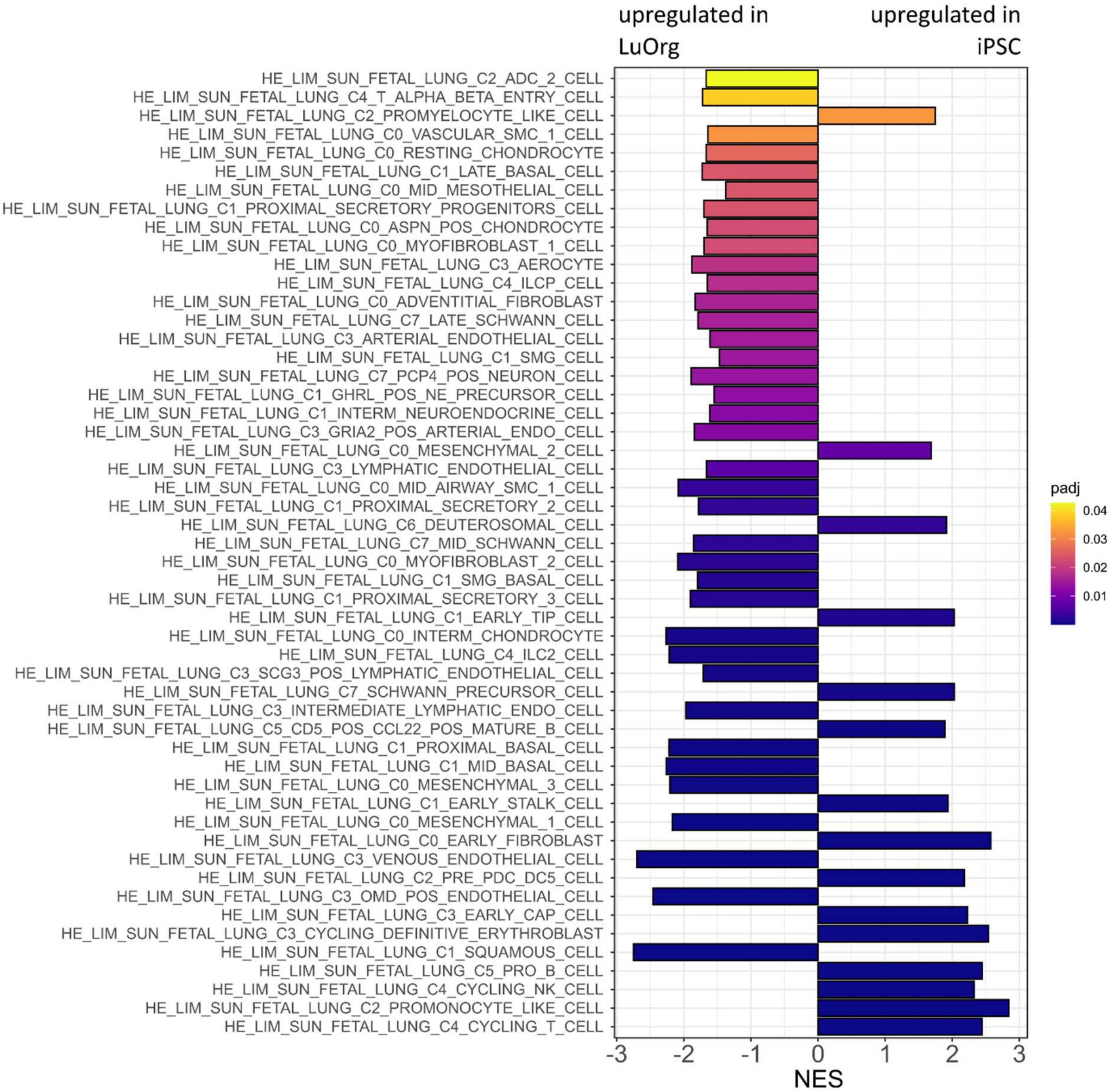

Molecular characteristics of bioreactor generated LuOrgs. Normalized expression scores (x-axis) and adjusted p-values (color) of the HE_LIM_SUN lung cell-type marker gene sets for significantly upregulated and downregulated genes using the bulk RNAseq data sets of generated LuOrgs and donor cells (iPSCs) depicted in Figure 2.

Supplemental Figure S4

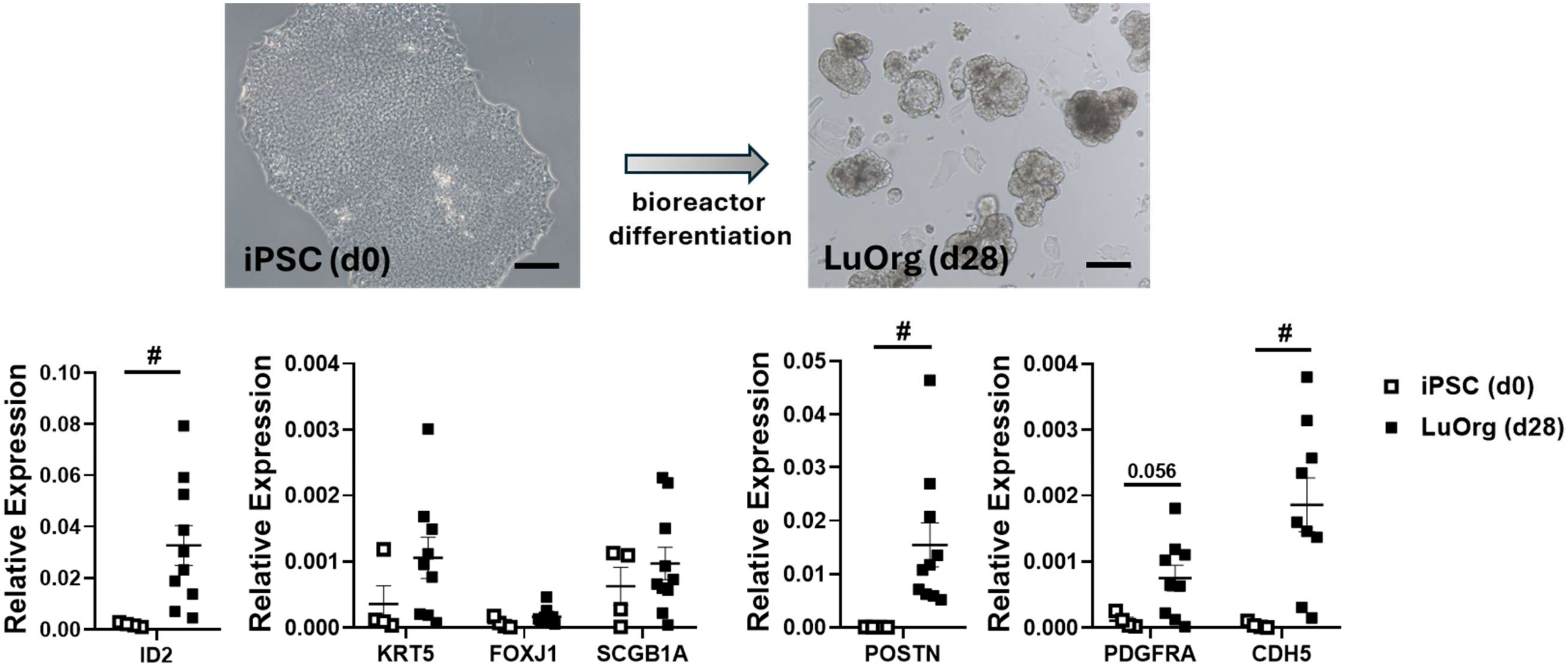

Molecular analysis of generated LuOrgs. Expanded iPSCs (d0) were harvested using an enzyme-free cell dissociation reagent and then directly transferred to the bioreactor (not shown). A representative phase contrast photograph is exemplarily shown. Magnification: 5x; scale bar 250 µm. LuOrgs generated from these iPSCs using the Bioreactor at the 28-day time point (end of experiment) are additionally shown. Magnification: 10x; scale bar 100µm. Indicated transcript levels were quantified using Real-Time RT-PCR and are shown as relative expression to beta-actin (set as 1). Biological replicates as indicated. The transcription factor ID2 (inhibitor of DNA binding 2) was used as marker gene for commonly distal structures, and the forkhead box transcription factor FOXJ1 was used for more proximal, bronchiolar cells. KRT5 was used as a marker for basal cells, and the gene for the secreted extracellular matrix protein POSTN (periostin), together with PDGFRA and CDH5 as mesodermal and endothelial markers. Secretoglobin family 1a member 1 (SCGB1A1) was included as gene encoding a pulmonary surfactant protein.

Supplemental Figure S5

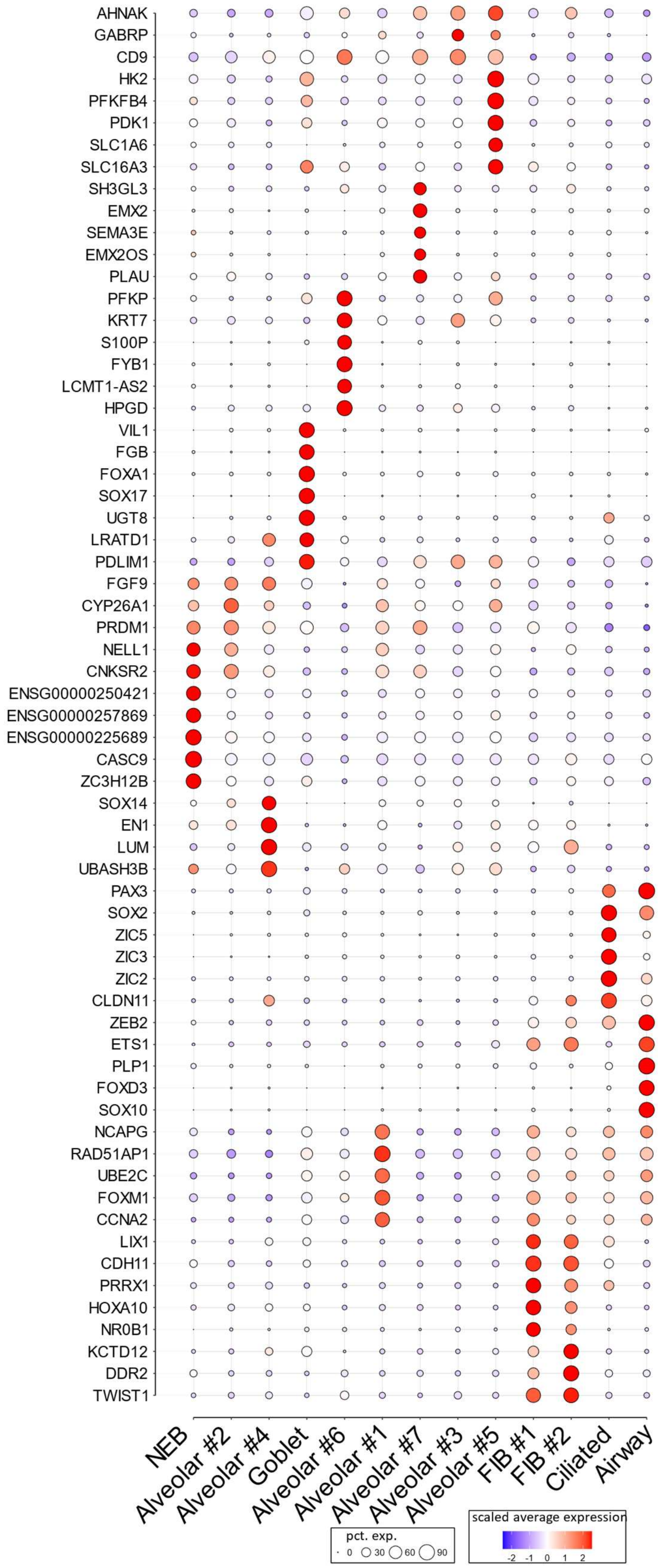

Single cell analysis of the composition of LuOrgs. Dot plot of top five differentially expressed genes per cluster. Size of the dots indicate the percentage of cells in which this gene was found, the color indicates the normalized value of the expression.

Supplemental Figure S6

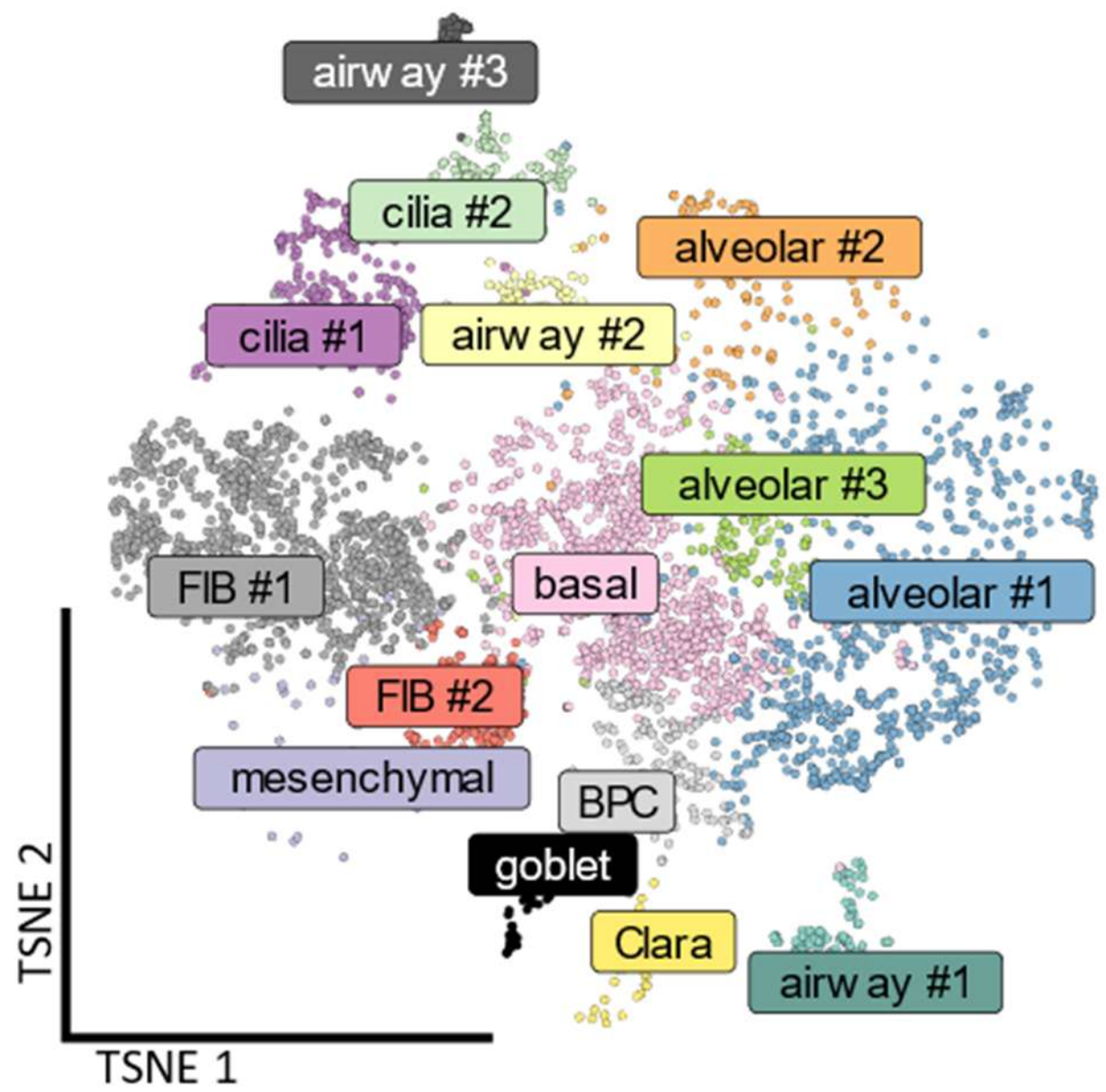

Clustering of the previously generated scRNAseq datasets from LuOrgs differentiated in the bioreactor from EBs. UMAP of all single cells colored by the identified clusters as shown in Front. Bioeng. Biotechnol. 2025;13:1684315.

Supplemental Figure S7

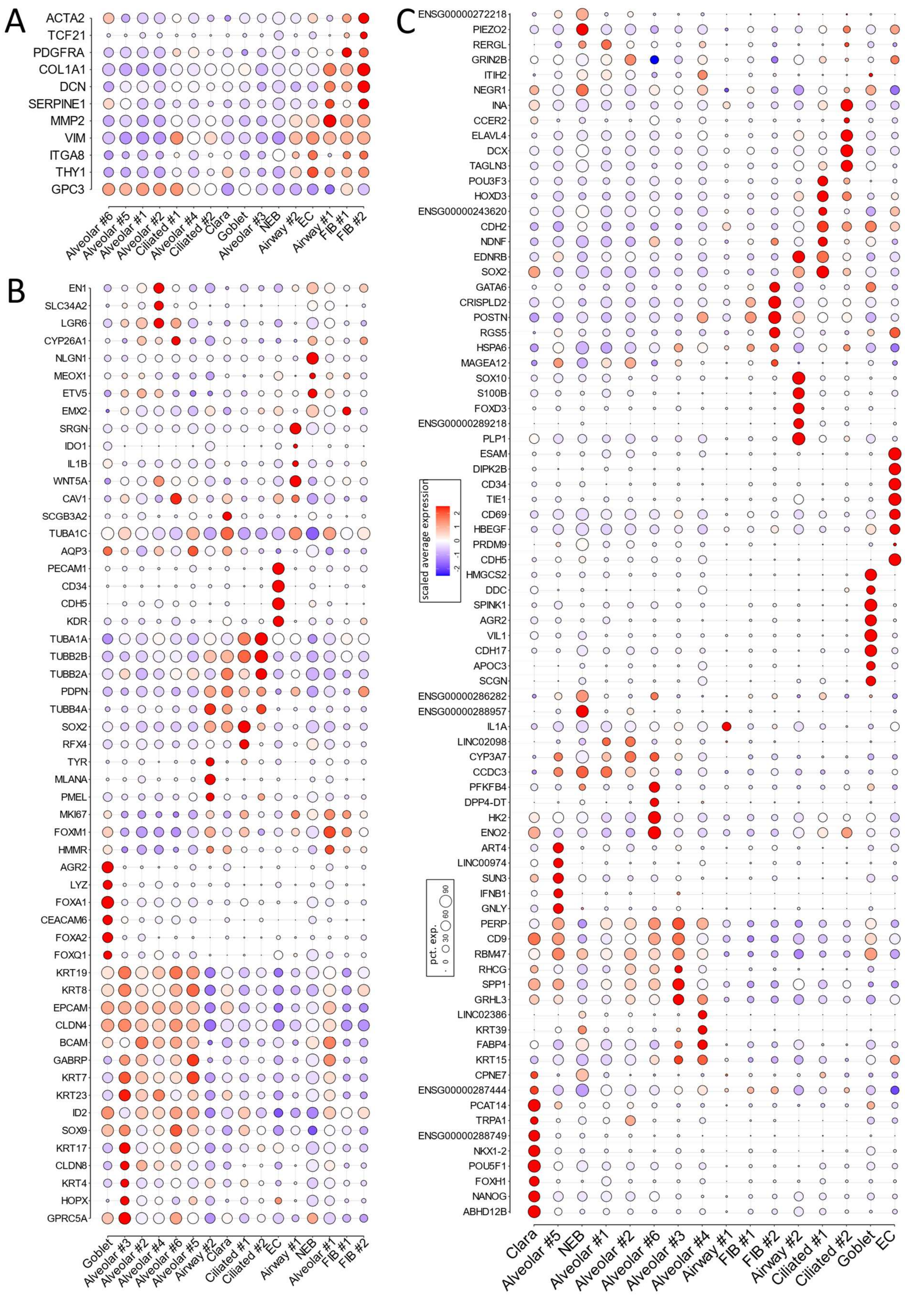

Combined clustering of the single cell RNA data sets. The scRNAseq datasets generated here from bioreactor-generated LuOrgs directly from iPSCs were compared with previously published scRNAseq datasets (Front. Bioeng. Biotechnol. 2025;13:1684315) from bioreactor-

generated LuOrgs derived from embryoid bodies (EB). Both single-cell datasets were re-clustered together (Figure 4). (**A**) Dot plot of indicated mesenchymal marker genes. (**B**) Dot plot of epithelial marker genes. (**C**) Dot plot of top five differentially expressed genes per cluster. Size of the dots indicate the percentage of cells in which this gene was found, the color indicates the normalized value of the expression.

Figure S8

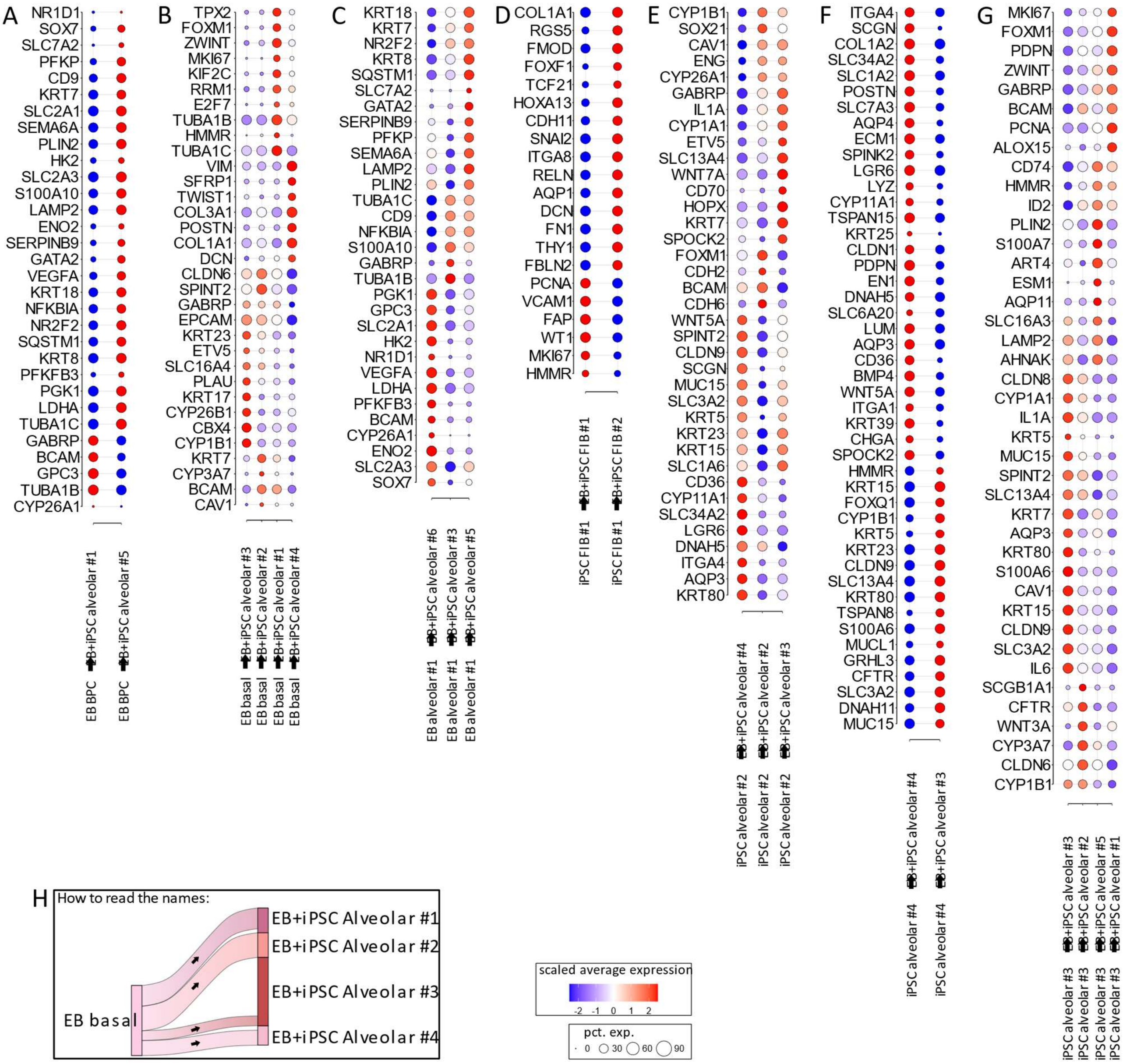

Association and abundance of clusters between EB-based, iPSC-based and combined LuOrg cells. Cluster sizes of the bioreactor-generated LuOrgs comparing EBs as starting material versus iPSCs as depicted in Figure 6. (**A-G**) Dot plots of subcluster identified by different allocation between the single dataset (EB-based or iPSC-based) versus the combination of both datasets. (**A**) EB-based BPC contains cells which can be allocated into two alveolar clusters. (**B**) EB-based basal contains cells which can be allocated into four alveolar clusters in the combined dataset. (**C**) EB-based alveolar #1 contains cells which can be allocated into three alveolar clusters in the combined dataset. (**D**) iPSC-based FIB #1 contains cells which can be allocated into both FIB clusters in the combined dataset. (**E**) iPSC-based alveolar #2 contains cells which can be allocated into three alveolar clusters in the combined dataset. (**F**) iPSC-based alveolar #4 contains cells which can be allocated into two alveolar clusters in the combined dataset. (**G**) iPSC-based alveolar #3 contains cells which can be allocated into four alveolar clusters in the combined dataset. (**H**) An example to help understand the labels is depicted.
